# Revealing administrative staff roles in primary care during the COVID-19 pandemic: a qualitative study of family physicians’ perspectives

**DOI:** 10.3389/frhs.2024.1471236

**Published:** 2024-11-26

**Authors:** Emily Gard Marshall, Lauren R. Moritz, Richard Buote, Maria Mathews, Julia Lukewich, Judith Belle Brown, Shannon Sibbald, Abraham Munene, Lindsay Hedden, Dana Ryan, Sarah Spencer

**Affiliations:** ^1^ Primary Care Research Unit, Dalhousie Family Medicine, Dalhousie University, Halifax, NS, Canada; ^2^ Department of Family Medicine, Schulich School of Medicine & Dentistry, Western University, London, ON, Canada; ^3^ Faculty of Nursing, Memorial University, St. John’s, NL, Canada; ^4^ Western University, London, ON, Canada; ^5^ Faculty of Health Sciences Simon Fraser University, Burnaby, BC, Canada

**Keywords:** primary health care, pandemic planning, health policy, family practice, delivery of health care, Canada, COVID-19, health care workforce

## Abstract

**Background:**

Administrative staff in primary care undertake numerous tasks to support patient care delivery. Although their roles are often overlooked, administrative staff are essential to the coordination and operations of primary care clinics. The COVID-19 pandemic introduced additional clinical and administrative tasks, including transitioning to virtual appointments and triaging patients for urgency, changing typical workflows. In Canada, existing pandemic plans for primary care did not account for these administrative tasks, nor the support that family physicians would require to continue to provide patient access to primary care. This research seeks to describe and understand the perceptions and experiences of family physicians of their administrative staff roles in primary care during the COVID-19 pandemic, to help inform future pandemic planning.

**Methods:**

We present findings from a qualitative case study across four regions in Canada: Vancouver Coastal health region in British Columbia, Ontario Health West region, the province of Nova Scotia, and the Eastern Health region of Newfoundland and Labrador. We conducted semi-structured qualitative interviews with family physicians (*n* = 68) across the four regions and thematically analysed the data.

**Results:**

We identified five salient themes in the data, including (1) applying public health guidelines, (2) educating patients on COVID-19 and COVID-19 services, (3) re-organizing patient visits, (4) maintaining adequate staffing, and (5) recognizing administrative staff contributions. During the COVID-19 pandemic, family physicians took on numerous additional roles to reduce the risk of transmission of the virus with the support of their administrative staff. Family physicians emphasized the challenges of maintaining adequate staffing, and the importance of administrative staff in enabling the provision of primary care.

**Conclusions:**

Existing pandemic plans do not account for increased administrative roles taken on by primary care administrative staff. Pandemic plans must include guidance for the roles taken on by primary care administrative staff, such as clinical tasks, as they will continue to play an important role in pandemic recovery. Supporting administrative staff would enhance primary care providers’ ability to manage care during pandemics, facilitate resilience, and decrease provider and administrative burnout.

## 1 Introduction

The COVID-19 pandemic forced marked changes in the typical workflow in primary care clinics. Family physicians (FPs) were responsible for transitioning from in-person to virtual care, communicating changes to patients, developing and enacting protocols for triaging in-person vs. virtual appointments and addressing novel logistical challenges (1–4). These changing roles came about quickly, in response to the rapid onset of the pandemic (5). Administrative staff, who are integral but often overlooked members of healthcare teams in primary care (6), supported FPs in taking on these additional roles.

“Administrative staff” in Canada are referred to as clerical or office staff, reception clerks, medical office or administrative assistants, medical receptionists, general practice receptionists, and front-desk staff, among a host of other names. Administrative staff are not recognized as a regulated or accredited profession but take on a variety of clinic tasks (7), including appointment scheduling, managing office supplies, communicating with specialists and pharmacists, and triaging the acuity of the patient for their visit (6–8). Administrative staff build relationships with patients, advocate to providers on behalf of patients, and help patients to navigate the health system (6, 9, 10). Administrative staff in smaller, fee-for-service clinics with fewer allied health professionals are more likely to provide more clinical roles such as triaging (9, 11, 12). These roles may have been heightened during the pandemic given additional pressures on health care providers. Given the concern over the FPs’ administrative burden, understanding and leveraging the contributions of administrative staff may help address primary care provider burnout (13, 14).

Pandemic plans in Canada inadequately accounted for the needs and roles required in primary care during a pandemic (5, 15) and, by extension, the needs and roles of administrative staff. Instead, guidelines were primarily targeted at secondary and tertiary healthcare settings (16). The purpose of this study is to describe and understand the perceptions and experiences of family physicians of the administrative roles in primary care during the COVID-19 pandemic, to help inform future pandemic planning and improve patient access to primary care in public health emergencies. Given that the administrative staff's primary role is to support the day-to-day functioning of the medical clinic, perspectives from FPs can help us to better understand their roles. Understanding the roles of administrative staff from the lens of the FPs is important in identifying opportunities and gaps that could improve patient access and care within primary care clinics as administrative staff increasingly make up a large portion of staff employed in primary care clinics (17) and would report to FPs. Our study may also offer insight into the supports FPs need to manage administrative and clinical work in primary care, and how other providers such as physician assistants and nurses can take on clinical work to relieve administrative staff and FPs.

## 2 Materials and methods

### 2.1 Study aims and design

We present findings from our qualitative case study conducted across four regions in Canada: the Vancouver Coastal health region in British Columbia (BC), Ontario (ON) Health West region, the province of Nova Scotia (NS), and the Eastern Health region of Newfoundland and Labrador (NL) (15). Overall objectives of this study included exploring the roles of administrative staff during a pandemic, and the barriers and enablers of fulfilling these roles, to inform future pandemic planning for primary care. Interview data included questions related to supports for FPs who were interviewed. The study protocol has been published elsewhere (15). Questions included the facilitators and barriers FPs experienced in performing proposed and actual roles during the pandemic, potential roles that FPs could have filled during the pandemic, as well as the facilitators and barriers to fulfilment; and how their gender influences their proposed, actual and potential roles as family physicians during the pandemic. Demographic information was also collected from participants (e.g., gender, years of practice, work settings, clinic roles) (15).

### 2.2 Participants

We recruited FPs to participate in semi-structured interviews via professional organizations, third parties, snowball sampling, newsletters, and social media (15). We recruited FPs across different demographic characteristics to achieve maximum variation in our sample (18). We recruited FPs with consideration to characteristics such as practice model, setting (e.g., community-based, hospital, residential care), location (e.g., urban or rural), and gender. In order to be eligible, FPs had to be licensed to practice at the time of recruitment, practicing or eligible to practice due to pandemic-related work plan changes, and practice in community, hospital or long-term care settings (15). Postgraduate medical residents, international graduates licensed to practice during the pandemic only, and FPs who do not take on clinical roles (i.e., are involved only in academic, research, or administrative work) were ineligible to participate.

### 2.3 Data collection and analysis

We conducted semi-structured interviews with FPs working in each region until data saturation was reached (i.e., no new themes were identified) (19). The semi-structured interview guide was informed by a previously conducted policy scan (5). Interviews took place over telephone or Zoom (20), were recorded and transcribed verbatim. Qualitative analysts from each region analyzed the transcripts using a robust, collaboratively developed codebook (15). The research team from each region developed a provincial codebook based upon early participant transcripts, which were amalgamated into a master codebook. The codebook was refined through cross-provincial coding exercises wherein each qualitative analyst coded one transcript from each province using the master codebook. We held meetings to discuss additions and changes to the master codebook until the primary investigator and all qualitative analysists were satisfied with the comprehensiveness of the codebook. We used qualitative description through a thematic framework analysis. Qualitative descriptive approaches facilitated a comprehensive summary of phenomena or events being studied with the context of those events (21). We managed study data using NVivo software (22). For this paper, we coded specifically for themes related to the roles of administrative staff and we analyzed the data using a thematic framework analysis which involves creating a matrix of cases (columns) and themes (rows), placing quotes and summaries of the data in the in-between “cells” (23).

### 2.4 Ethical clearance

This study was approved by Research Ethics British Columbia (No. H20-02998), Western University Research Ethics Board (Project ID 116315), Nova Scotia Health Research Ethics Board (File No. 1026085), and Health Research Ethics Board of Newfoundland and Labrador (No. 2020.251).

## 3 Results

We interviewed 68 FP participants across the four regions. Table 1 details participant demographic information. Demographic information was collected on participants location, gender, years of practice, work settings, clinic roles and whether they had dependents.

**Table 1:** Demographic summary of family physician participants (*n* = 68).

Variable	*n* (%)
Province	
British Columbia	15 (22.1)
Ontario	20 (29.4)
Nova Scotia	21 (30.9)
Newfoundland and Labrador	12 (17.6)
Gender^a^	
Man	27 (39.7)
Woman	41 (60.3)
Practice type	
Alternative payment model^b^	39 (57.4)
Fee-for-service	21 (30.9)
Blended	8 (11.8)
Years in practice (mean, range)	16.8 (7 months–38 years)
Hospital privileges^c^	
Yes	49 (72.1)
No	19 (27.9)
Rurality	
Rural	20 (29.4)
Urban	44 (64.7)
Small urban	1 (1.5)
Mix	3 (4.4)
Care for dependents^d^	
Yes	42 (61.8)
No	24 (35.3)
Not stated	2 (2.9)

^a^ Gender was asked as an open-ended question.

^b^ Alternative payment includes all non-fee-for-service or enhanced fee-for-service payment types. Blended payment includes providing primary care in different settings where payment is fee-for-service in one and alternative in another.

^c^ Physicians who were allowed to admit and see patients in hospital or the emergency department and refer patients for hospital-based lab and diagnostic services.

^d^ May include children, older family members, etc.

**Figure 1:**
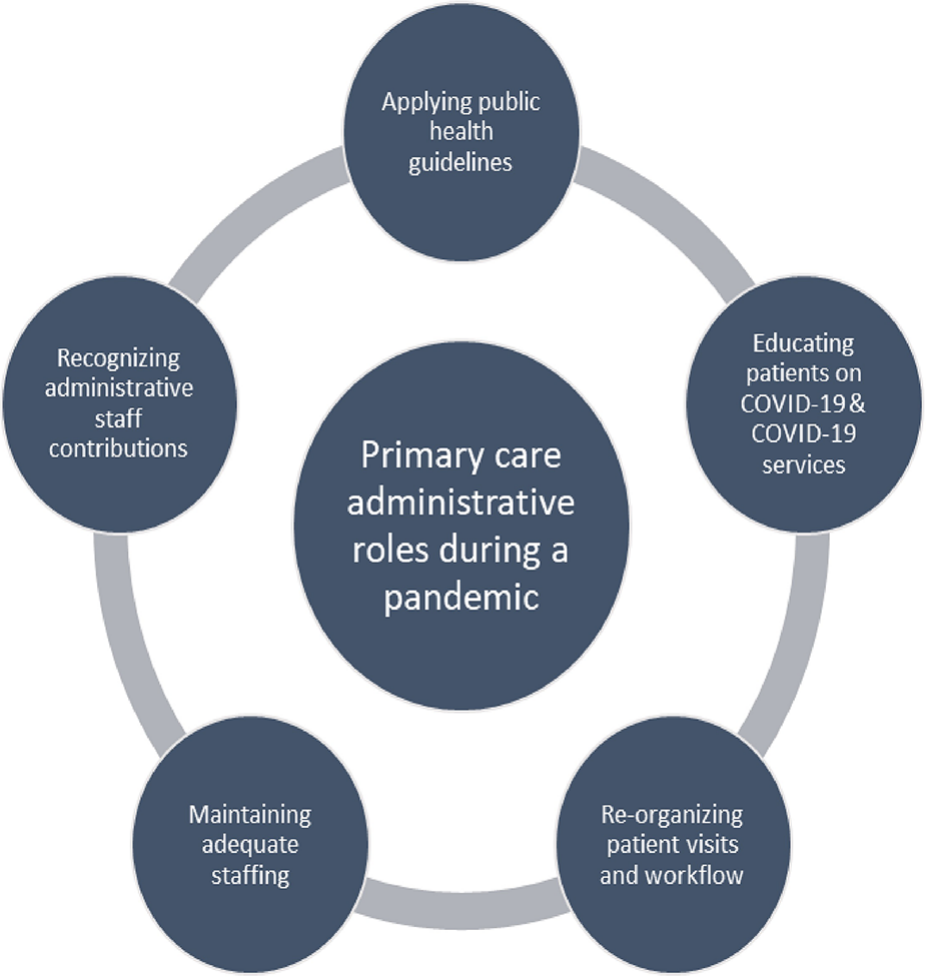
Primary care administrative roles during a pandemic.

Salient themes relevant to family physicians and their administrative staff during the pandemic include: (1) applying public health guidelines, (2) educating patients on COVID-19 and COVID-19 services, (3) re-organizing patient visits, (4) maintaining adequate staffing, (5) recognizing administrative staff contributions (Figure 1).

### 3.1 Summary of themes relevant to family physicians and their administrative staff

Administrative roles were often fluidly enacted by or through FP and administrative staff. FPs often developed public health guidelines that were implemented by administrative staff. FPs often sourced information that was shared with patients by administrative staff. FPs worked with administrative staff to decide how to manage patient visits. FPs were responsible for maintaining adequate staffing, approving leaves of absences, and protecting administrative staff from burnout. FPs recognized the importance of administrative staff in the provision of primary care.

#### 3.1.1 Support for applying public health guidelines

Administrative staff supported FPs in operationalizing public health protocols such as active and passive screening for COVID-19 symptoms: “*the receptionist just asks the patient to read [the list of screening questions] and answer the questions. And she checks their temperature”* [BC, fee-for-service (FFS)]. A participant from NS shared that administrative staff were “on the ball” with screening patients:

“two patients… were symptomatic and they didn’t meet the Public Health screening criteria but… our front desk staff is super on the ball, and she called… and said, “What do you think?” And I said, “Just put them in the isolation room, you know, it sounds a bit suspicious.” And then they ended up having COVID” (NS, alternative payment plan ).

Some FPs shared that administrative staff were also “*cleaning when they normally wouldn*’*t be doing that much work from a cleaning perspective”* (NS, blended).

Administrative staff also helped to take “*inventory… of our PPE supply”* (BC, APP) and did “*some… tricky maneuvering* (organizing patient flow) *to make sure there was never more than a few people in the wait room at a time”* (NS, FFS).

Administrative staff were often shouldering the brunt of backlash of challenges in implementing new public health policies as they emerge:

“…to not be able to easily get answers on how to do things, that to me was… an unnecessary frustration… we would… talk with… the manager of this department (public health department) about things, and they’d say, “Oh, no, you need to do this.” And then… we would talk with someone else, and, “No, no, you need to do this.” And… people would… send back… test requests, and be very frustrated, yelling at our front desk because we weren’t doing it properly. And we were trying. We were just trying to do what the previous person told us to do. So, you know, I guess at a local level, having clarity around the processes of providing care, I do not think that went well at all. It was very stressful. And again, things were always changing” (NS, APP).

Administrative staff operationalized public health protocols such as patient screening and cleaning tasks, and managing patient flow that were important within the clinical setting. However, these task may not have been perceived as an appropriate use of time or energy by FPs or minor in relation to their patient care role. Furthermore, as front facing staff and first point of contact for patients and public health officials, administrative staff may have bore the brunt of frustrations of patients and public health enforcers.

#### 3.1.2 Contribution to educating patients on COVID-19 and COVID-19 services

Administrative staff played a role in communicating with patients and answering their questions pertaining to COVID-19 and COVID-19 care. They were often the first point of contact for general questions: “*A lot of patients were scared and worried, and they would call us. And our poor admin staff got asked more strange questions last year that they obviously wouldn't know how to answer”* (NS, blended). Administrative staff also answered questions about COVID-19 testing “*We were referring people on to 811 for testing… we had the resources we needed from the information given out by the health authority on that… almost daily bulletin update.”* (NS, APP) as well as about COVID-19 vaccination: “*… before the vaccination schedule [was] announced… patients were calling the front [desk], saying… “Can I get on the list?”* (BC, APP); as well as COVID-19 testing.

FP participants also noted their administrative staff's role in communicating public health protocols to patients: “*when patients phoned..the admin. Staff were the ones who kind of let the patients know what the new normal was at the time and they would tell people as they phoned.”* (NS, APP); In managing appointments, administrative staff also assumed the task of explaining the need to prioritize urgent visits: “*We did have our administrative staff communicate that to patients… “We’re here. We have limited time. Really preference is to be seeing acute or new issues only at this time. Having said that, what do you need?””* (NS, FFS).

As front facing staff, administrative staff were put in a position to answer questions from patients regarding COVID-19, even if they may have not known the answers given the novelty of the pandemic situation. Some FPs may have felt sorry for their administrative staff as they perceived them not having the knowledge to answer patients’ questions. Administrative staff often had to refer patients to public health information lines (e.g., 811) and convey new public health measures to patients.

#### 3.1.3 Re-organizing patient visits and workflow

FPs made many changes to how patient visits were organized, with support from their administrative staff who assisted with “*developing schedules… for our clinics”* (ON, APP) and “*shuffling people around to accommodate the schedule of what I could do in the get-go..”* (NS, FFS).

FPs had to prioritize patients for urgency and in-person visits, with the support of their administrative staff:

“… when [patients] called in to make the appointment… if our receptionist thought it was something that needed to be seen in person right away, she would just book it. Otherwise, we would do the phone call first and then if we thought we needed to see them in person we would just book them for our… next slot” (ON, APP).

Some interviewees found that their “*admin staff became very skilled at deciding who needed to come in and who didn*’*t… right away”* (NS, blended), however, other FPs found this task was “*a bit too clinical for the admin person to do”* and preferred to have “*one physician and one allied health staff… triaging and managing phone calls”* (BC, APP).

According to one FP whose practice was located in a hospital, administrative staff supported patients with booking their lab and diagnostic imaging appointments as the province implemented online patient booking systems:

“… before patients started booking themselves for lab and x-ray, the hospital administration was helping with scheduling people for lab and x-ray, and so they actually kind of started to help our clinic a little bit, help our patients with lab and x-ray. So, we actually got some admin staff from the hospital… helping us out with things like that so there was a benefit there… in getting a little bit of extra help from the hospital” (NS, APP).

With the changes to virtual care billing codes, administrative staff also had to learn how to bill for virtual visits:

“… there was a bit of confusion at the beginning, what could you bill, how could you bill, what did you have to document for time and… phone visit and all that… luckily that got settled fairly quickly… the billing codes seemed to be quite easy for the secretary, to just continue to bill, simply put in that it’s a telephone visit, or a virtual visit” (NS, APP).

With the move to virtual appointments, administrative staff and providers alike were figuring out how to work from home with some challenges and limitations: “*Front desk, a lot of it*’*s on the phones so, trying to find ways even for them to do it from home”* (ON, APP).

Some participants found that having an electronic medical record was useful for enabling them to communicate with their administrative staff “*… the electronic medical record… it makes it so easy because… there*’*s not really much difference being in the clinic… or being in your house, because you can communicate through the electronic system with the receptionist…”* (NL, APP).

Participants also identified innovations that could help relieve some of the workload of their administrative staff such as online booking, which was not available at the time *“… why don't we have online booking? Like, people can use that, and then it would take some of the work off of the front desk”* (BC, APP).

Administrative staff played an important role in organizing patient flow. Administrative staff may have been given some autonomy in booking patients. However, FPs may have felt this task may have overreached onto their clinical expertise and opted to do it collaboratively. Administrative staff also had to learn new technologies (e.g., virtual care), that were used in lieu of in person visits and familiarize themselves with how these services were billed.

### 3.2 Human resources considerations

Given the important functions administrative staff were doing in supporting public health guidelines, educating patients about COVID protocols and procedures, and organizing patient flow, FPs shared how important it was to retain their staff and recognize the work they do for their practices to function effectively.

#### 3.2.1 Maintaining adequate staffing

Several FP participants shared concerns about staffing. FFS FPs in particular dealt with reduced clinic revenue as patient volumes dropped, as well as reported challenges in retaining administrative staff:

“… I tried to keep my [administrative staff] employed, but after a while it was just impossible… I had to lay her off and she took [Canada Emergency Response Benefit] payments for a few months and… she hopes that I can reopen and rehire her; she’s… an excellent [administrative staff member], but I can’t even afford to pay her now… I am literally hanging on with my fingernails” (BC, FFS).

Administrative staff were also impacted by lack of childcare:

“… when closure happened, [administrative staff member] had to be home with her two-year-old… there isn’t like a [human resources] agency for primary care… where we could call up and say, “Hey, I need a receptionist who knows this [electronic medical record] to come and fill in for three days or whatever,” we don’t have that” (ON, APP).

Participants expressed concerns that administrative staff would leave their role due to the fear of COVID-19 exposure: *“We had to deal with staff leaving because of the pandemic. I had a secretary say, “It's too much for me, I just can't work in healthcare anymore.” So that was a huge stress… we said to her… “Take a leave of absence.” She just said, “It's too anxiety-provoking for me because of my mother's health, I can't bring it home”* (BC, FFS).

Challenges to retaining staff included the lack of funding to pay staff due to decreased revenue in FFS clinics, the lack of adequate support for staff members who had families to take care of and the high stress environment that resulted during the pandemic. Given these challenges, FPs felt that ensuring that administrative staff were recognized and felt supported was necessary for retaining their administrative staff.

#### 3.2.2 Recognizing administrative staff contributions

FP participants described the importance of having the support (e.g., informational and organizational) of their administrative staff. Several FPs described their administrative staff as “*the most supportive of all”* (NS, FFS) and “*the backbone of being able to allow physicians to… deliver their care*” (NL, APP).

Some FPs found it challenging to work from home without the support of their administrative staff:

“… I worked from home for maybe 2 or 3 weeks… it’s so hard because you’re your own receptionist and you’re faxing prescriptions and then the technology doesn’t work and… it was a nightmare. And… I found that I was more overwhelmed being at home” (BC, APP).

Despite their importance, FPs pointed out that role of administrative staff was overlooked in the pandemic planning and response:

“… all of the organizations (public health organizations and health authorities), they target their communication to the physician, and they completely neglect our office staff… physicians were having to pore over that with their office manager and you have their secretary (primary care administrative staff) asking them questions” (ON, APP).

FPs described steps taken to support their administrative staff, including strategies to promote psychological safety:

“… if [administrative staff] leave, we’re done. So, we had to make sure that they were comfortable… at one point they were using the cheaper masks, and you hear them complain and you’re like, “No, you guys are in the front, you guys pick whatever masks you like. Here’s the selection.” I can live with whatever, I’m so used to wearing a mask, but that’s not the life they chose. They’re receptionists, they’re medical office admin… this wasn’t necessary a vocation in the same way as it is for us… we have staff who may not feel quite as enthusiastic about being in the thick of it or… being on the front line” (NL, FFS).

FPs also recognized the need to “check-in” with their teams, including administrative staff:

“… the importance of regular staff meetings and not just covering… the how do we function and what are the process issues. We now actually spend time and go around and ask each other, how are you doing? We do a mental health and physical health check-in. What’s going on? That idea of being very mindful of each other’s health” (NS, APP).

FPs shared the importance of including administrative staff in decision-making:

“When you quickly move from face-to-face patient to all telephone… You know, you need input from your staff to say this is what we’re going to do. Who mans the telephone? What if you need three people to man the telephone because the call volume is so high? How do you do that? So, by having them engaged in the process, that fact that they’re doing something and doing something productive, providing that added patient care also lends itself to reducing anxiety” (NS, APP).

FPs felt that administrative staff were an essential “*backbone*” to their practices and may have found it difficult to navigate the administrative duties that they may not have been regularly accustomed to. FPs also noted that public health guidelines often overlooked administrative staff even though they were the first point of contact with patients. FPs were also concerned about the safety of their staff during the pandemic and felt that ensuring they had the right equipment and psychological support for them to carry out their duties was necessary to ensure their practices run effectively. FPs also stated that incorporating their staff in decision making was important in recognizing their abilities and perspectives, even though knowledge and power imbalances in terms of who is responsible for clinical roles may have been present.

## 4 Discussion

The COVID-19 pandemic created a need for rapid shifts in primary care delivery, resulting in new roles for FPs and their administrative staff. In this study, we sought to understand the roles of administrative staff within primary care from the perspectives of FPs, to help inform future pandemic planning. Over the course of the COVID-19 pandemic, the work of administrative staff had direct implications for patient access to primary care and the safety of primary care providers and their patients.

In addition to their normal responsibilities, administrative staff were directly involved in providing support in applying public health guidelines. The FPs we interviewed shared how their administrative staff became responsible for ensuring patients were screened for COVID-19 symptoms, were informed about COVID-19 and pandemic services, and were prioritized for in-person or virtual care, often without guidance or resources. Administrative staff took on these roles in the face of a public health emergency and functioned as a critical part of the work teams in primary care, often without protection, training, and adequate supports. FP participants dealt with significant challenges when their administrative staff needed to take leaves of absence due to stress, childcare responsibilities, or to isolate following COVID-19 exposure or infection. Participants emphasized the importance of supporting the mental health of their administrative staff. FPs identified potential supports to remove some of the burdens from administrative staff such as use of electronic medical record systems to substitute in-person communications and allow their administrative staff to work from home. Other supports include the use of asynchronous virtual care modalities which include automatic release of lab results to patients and direct patient appointment booking, alleviating administrative staff of these responsibilities (24). FPs emphasized the importance of administrative staff and working collaboratively to enable the continuation of primary care provision and ensure resilient health systems.

FPs also identified that administrative staff took on a significant role in educating patients on COVID-19 and services offered during the pandemic. This role of education was taken on despite administrative staff having no formal education in areas such as disease prevention and control. Prior to the COVID-19 pandemic, researchers noted that increased demands on primary care providers have not resulted in corresponding changes in training for administrative staff (25, 26). Our findings point to the potential need for additional training opportunities for administrative staff in primary care which may allow them to better carry out the tasks that are asked of them, particularly during a pandemic. Having interdisciplinary training and collaborative learning opportunities between administrative staff and healthcare providers may be beneficial. Additionally, the training and pay scales for administrative staff should be reviewed and may require additional supports and funding to meet the needs for primary care access during pandemics.

Re-organizing patient visits for either in person or virtual care became an important task during the pandemic FPs used their own professional judgement to delegate tasks to administrative staff, but some tasks, like assessing acuity of patient needs, were sometimes “too clinical” for administrative staff. During the pandemic context, FPs reported relying more on administrative staff to work outside of their typical scope and training (e.g., triaging patients, which is a clinical skill they are not trained in). Decision-makers may wish to consider the appropriateness of administrative staff being delegated this work, and whether additional training that is interdisciplinary in nature, regulation, or public health support is needed during pandemics. Training administrative staff to provide such clinical activities within the context of primary care team could help to unburden other providers (26), but strategies will be needed to account for the increased administrative staff workload and remuneration expectations.

Maintaining adequate staffing levels during the pandemic was an important issue discussed by FPs. This was particularly noted by FPs operating on a FFS remuneration model. This was because FFS clinics that may have relied on billings generated from patient visits saw a decline during the pandemic (27). Participants who were paid using a FFS remuneration model were more likely to face financial challenges compared with non-FFS FPs, which could result in the need to lay off administrative staff during the pandemic. Although FFS FPs in some Canadian provinces were offered financial supports to minimize hardship during the pandemic (28–30), our participants found that these were not always sufficient. Primary care administrative staff need to be included in pandemic planning documents. This could include, for example, provisions for ongoing funding of administrative staff for FPs who pay their administrative staff’s salaries to ensure continued staffing when clinical volumes (and thus provides’ incomes) are low. It could also outline the specific roles for administrative staff in enacting pandemic planning policies. Although participants did not share the gender of their administrative staff, previous research has found that administrative staff are largely women (10, 31). Future research could explore how the gendered nature of childcare responsibilities during a pandemic impacted primary care workforce, including administrative staff (32).

FPs spoke about human resource challenges and opportunities while recognizing that administrative staff were essential to the effective functioning of the family practice. Providing additional supports to improve the work conditions of administrative staff (e.g., mental health support, check-ins, and time off for family responsibilities including childcare) and increasing collaboration between administrative staff and clinicians was viewed by FPs as a way of supporting and acknowledging the work of administrative staff. Furthermore, to alleviate some of the stressors faced by physicians within their practice and potentially reduce some of the clinical tasks that may fall onto administrative staff, some provinces in Canada have introduced physician assistants in primary care settings. Recently, NS became the fourth province to introduce a training course for physician assistants (33). Physician assistants are trained to provide clinical support to physicians in a variety of medical settings, including primary care. Our findings may point to the need for other healthcare providers to step in and provide clinical support that is beyond the scope of administrative staff.

### 4.1 Strengths & limitations

The findings from this study reflect FP perspectives on the roles of their administrative staff across four provinces in Canada, describing experiences and systemic issues that were often similar across provinces. We did not directly interview administrative staff, rather we sought perceptions and experiences of FPs on administrative roles. Administrative staff may have described their roles differently. However, this is the first study we are aware of examining the roles of the administrative staff during a pandemic or public health emergency and future research should include administrative staff as participants to improve understanding of the complex roles they took on during the pandemic. The many roles taken on by administrative staff are often not well understood by their colleagues (29), and their first-hand perspectives may offer an in-depth understanding of the conversations they had with patients, the tasks they took on as part of the pandemic response, and the supports they needed in their roles. We made efforts to recruit physicians with varying demographic profiles and many of our participants were urban dwelling, women, and physicians with dependents. The proportion of female physicians who participated in our study is slightly higher (60.3%) than the proportion of female FPs in Canada (49.7%) in 2022. Their lived experience as female physicians with family members to look after may have influenced some of the perceptions presented during interviews. We conducted the study in four provinces. This is a strength of the study and increased the variability of the responses we received that informed our themes, thus adequately capturing FPs experiences across the primary care landscape in Canada. However, we recognize that the differences in geographical locations, practice models, and practice settings may have influenced some of the responses. For example, different provinces may have had differences in the way they enforced COVID-19 measures and how stringent they were in adopting federal guidelines. Furthermore, practices may adopt funding models that work best within their locations and jurisdictions taking into consideration legislation, their patient population, and available resources.

## 5 Conclusion

The purpose of this study is to describe and understand the perceptions and experiences of FPs of the administrative roles in primary care during the COVID-19 pandemic. Understanding the work procedures, relationships, and communication between FPs and administrative staff is important for achieving good quality service and patient care. These roles and professional relationships may be elucidated by exploring the perspectives of one group about the other. FPs described administrative roles carried out within their practices by administrative staff. During the COVID-19 pandemic, the work of FPs with their administrative staff had direct consequences for patient and provider safety, access to primary care, and the processes required to enable that access. Despite their critical roles, existing pandemic plans do not account for the responsibilities of administrative staff. Steps should be taken to prepare pandemic plans in advance that include guidance for the roles taken on by primary care administrative staff, as they will continue to play an important role in pandemic recovery and future public health emergencies. Attention to supporting administrative staff would enhance primary care providers’ ability to manage care during pandemics, facilitate resilience, and decrease provider and administrative burnout. Consideration must also be given to potential shortages of administrative staff during a pandemic and what contingencies are necessary to ensure the needs for primary care during pandemics.

## Data Availability

The raw data may be available upon request if the request meets the funding organization and confidentiality requirements of the participants data.
